# Ecological Health Risk Assessment and Source Identification of Heavy Metals in Surface Soil Based on a High Geochemical Background: A Case Study in Southwest China

**DOI:** 10.3390/toxics10060282

**Published:** 2022-05-25

**Authors:** Ziwan Chen, Jing Xu, Ruichun Duan, Shansong Lu, Zhaolei Hou, Fan Yang, Min Peng, Qingxia Zong, Zeming Shi, Linsong Yu

**Affiliations:** 1 School of Earth Sciences, Chengdu University of Technology, Chengdu 610059, China; sean_yls@163.com; 2 Applied Nuclear Technology in Geosciences Key Laboratory of Sichuan Province, Chengdu University of Technology, Chengdu 610059, China; 3 Department of Geophysical and Geochemical Exploration, Yunnan Institute of Geological Survey, Kunming 650216, China; yj_890209@126.com (J.X.); h372420685@163.com (Z.H.); qingxiazong@163.com (Q.Z.); 4 Wuhan Center, China Geological Survey (Central South China Innovation Center for Geosciences), Wuhan 430205, China; duanruichun_123@163.com (R.D.); luyin_student@163.com (S.L.); 5 Institute of Geophysical & Geochemical Exploration, Chinese Academy of Geological Sciences, Langfang 065000, China; yf51318@163.com (F.Y.); pmin@mail.cgs.gov.cn (M.P.)

**Keywords:** heavy metals, high geochemical background, potential ecological risk, human health risk, PMF, lead–zinc mine

## Abstract

A total of 28,095 surface soil samples were collected in areas with high natural background levels; the potential ecological risk is generally low, and the high-risk area is small and mainly affected by lead–zinc mines. The contribution to the potential ecological risk factor (*RI*) is as follows: Hg > Cd > As > Pb > Cu > Ni > Cr > Zn, with noncarcinogenic chronic risks of Cr > As > Cd > Pb > Ni > Cu > Hg > Zn; furthermore, dermal contact is the main pathway of exposure causing health risks. The total carcinogenic risks caused by heavy metals were as follows: Cr > Cd > As > Pb; and the risks posed by Cr, Cd, and As were higher than the threshold value (1.0 × 10^−4^); people face a higher threat to heavy metals in soils in Zhenxiong, Ludian, Huize, Weixin, and Zhaoyang. The evaluation result of the EPA PMF model shows that the soil heavy metals are mainly composed of five sources, of which basalt, Permian, and Triassic carbonate rock parent material constitute the natural background source, while the mining activities of lead–zinc mines and the emissions of coal burning by residents constitute the anthropogenic source. The contribution was ranked in order of lead–zinc mining (26.7%) > Triassic carbonate (23.7%) > basalt (20.9%) > coal burning and automobile emissions (16.1%) > Permian carbonate (12.6%).

## 1. Introduction

Ecological risk assessment is an important part of environmental risk assessment, and it is the process of assessing the possibility of negative ecological effects occurring because of exposure to single or multiple pollution factors [1]. Health risk assessment, including noncarcinogenic and carcinogenic risk assessment, has been recognized as an important tool for identifying health risks in human activities [2]. To date, several studies have been carried out on the ecological risk assessment of heavy metals in contaminated soil [3,4,5]. In recent years, the ecological-health risk assessment of heavy metals in soil has mainly focused on the safety of polluted mining areas, enterprises, and agricultural soil or crops [6,7,8,9,10,11,12,13]. These areas have obvious human activities, and the research results are based on the evaluation of the total amount of heavy metals and the complete exposure pathways. However, there are few studies on heavy metals in soil from nonagricultural or nonindustrial areas; these sites are considered to be a source of natural metals, and the influences of soil from different land-use types are rarely emphasized in the process of risk assessment.

Northeast Yunnan is a typical area with high geochemical levels of heavy metals in southwestern China. Compared with the background value of soil (layer A) in China [14,15], there are eight heavy metals of interest, including arsenic (As), cadmium (Cd), chromium (Cr), copper (Cu), mercury (Hg), nickel (Ni), lead (Pb), and zinc (Zn); these eight heavy metals show enrichment, except for As, Cu, and Cd, which are strongly enriched, and their enrichment coefficients are 3.2 and 5.8, respectively. Northeast Yunnan is a region with a high geochemical background of heavy metals in soil. Cheng et al. [16] believe that the parent material in the area is the decisive factor affecting the composition of elements in the soil. Some studies suggest that the natural source of heavy metals in the soil is caused by the weathering parent mafic rocks, black shale, and limestone [17,18,19,20]. The clay minerals and Fe–Mn oxides in basalt weathering products are the main reasons for the enrichment of Ni, Cr, and other heavy metals in surface soil [20,21,22]. Wang et al. [20] traced the source and migration process of heavy metals through the basalt-surface soil-crop system using Ni in the basalt weathered soil of eastern China for the first time. Wu et al. [23] carried out a large-scale ecological risk assessment on agricultural soil by using a regional evaluation model and tried to discuss the relationship between the assessment results of the whole region and the parent material of the soil, and the results showed that the large-scale evaluation depended largely on the analysis of the parent material. Wen et al. [19] believed that the heavy metals in soil with a high geochemical background came from the weathered accumulation of the parent rock based on the isotope analysis of the soil profile; however, they have not completed the classification of natural heavy metal sources. Northeastern Yunnan is a typical middle-alpine cutting landform, with scattered and diverse land-use types, and the range of high geochemical background levels of heavy metals in the soil is large. Relevant research shows that there is a close relationship between ecological risk and land-use type [24,25,26,27,28]; thus, risk assessments that do not consider land-use type may overestimate or underestimate the risk level of heavy metals in the soil, resulting in excessive treatment costs.

Basalt and carbonate rocks are widely distributed in the area with high geochemical background levels in northeastern Yunnan, and those rocks have become the main soil-forming parent materials. The special geographic location and topography determine the diversity of land-use types in the study area. Therefore, these factors cannot be ignored when conducting soil ecological geochemical assessments in the study area; specifically, it is believed that the heavy metal sources of soil are caused by different land-use types and different parent materials. The results of the comprehensive classification of soil environmental quality show that the first-class environmental quality of soil in northeast Yunnan spans an area of 1456 km^2^, accounting for 5.2% of the survey area, and the second-class quality spans 86.5% of the survey area, which shows that the heavy metals in the soil in the area pose a high potential risk. It is of great significance to carry out surveys of heavy metals in soil and ecological health risk assessments in northeast Yunnan.

This article refers to a relatively mature risk assessment model in the “Ecological Risk Assessment Guide” from the US Environmental Protection Agency (USEPA), combined with the land-use types of the study area. The research focuses on eight heavy metals, including As, Cd, Cr, Cu, Hg, Ni, Pb, and Zn, and the risk of chronic intake and carcinogenicity of soil in the high geochemical background area of heavy metals of northeast Yunnan were evaluated under three exposure methods: dietary intake, unintentional inhalation, and dermal contact. At the same time, the positive matrix factorization model (PMF) proposed by the USEPA [29] was used to identify the source of heavy metal pollutants in the high geochemical background area in a large spatial range. The regional ecological risk assessment provides examples and assistance for the management of heavy metals from natural sources and the selection of research methods in typical areas with high geochemical background levels of soil heavy metals in southwest China.

## 2. Materials and Methods

### 2.1. Research Area

**Figure 1:**
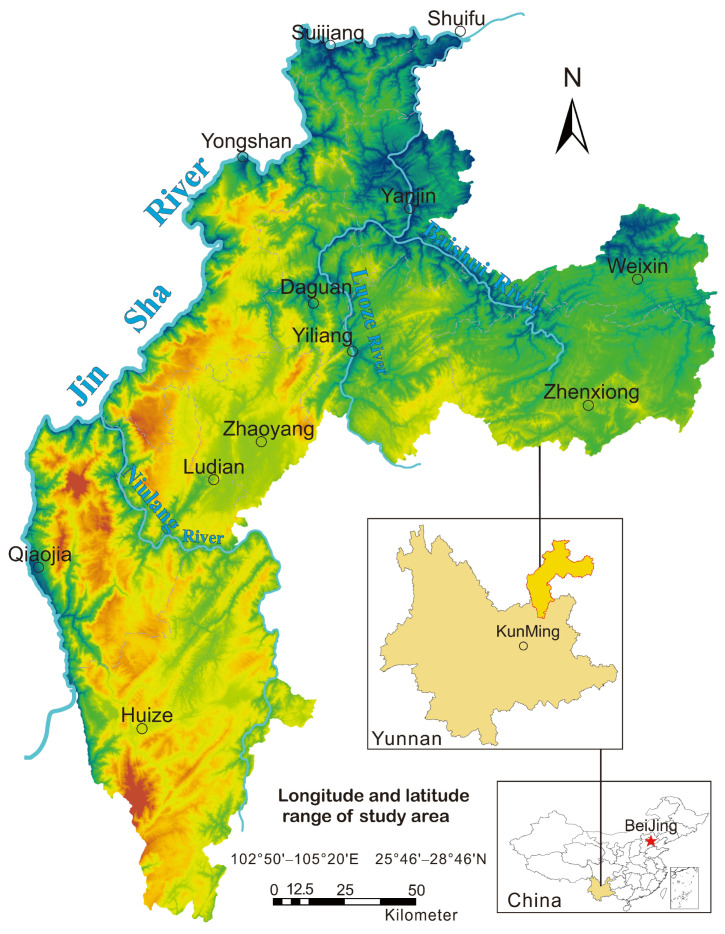
Location map of the study area.

The study area is located in northeastern Yunnan Province, southwestern China. The geographical coordinates are 102°50′–105°20′ E, 25°46′–28°46′ N, covering approximately 28,066 km^2^. The administrative division is under the jurisdiction of Zhaotong and Qujing (Figure 1). The study area has a plateau monsoon three-dimensional climate with both plateau subtropics and warm temperate zones, with an average annual temperature of 12.6 °C. The highest altitude in the area is 4025 m, and the lowest altitude is 175 m, which belongs to the deep-cut alpine landform, and most of the area is composed of carbonate karst. Various types of land resources are scattered in the area because of the influence of topography and landform diversity, and the land-use types are diverse and complex. The predominant land-use types in the study area are forestland and grassland, accounting for 58.6% of the total land area, while agricultural land accounts for only 35.5% [30]. The area is mainly based on agricultural planting, and the special landform provides unique conditions for agricultural production in the area; at the same time, the unique landform necessitates higher requirements for adapting agricultural production to local conditions. The research area is located in the middle and upper reaches of the Yangtze River and is located in the watershed area of the Jinsha River, the Niulan River, and the Luoze River, with other water systems running through it. It is an important soil and water conservation area. In recent years, the ecological environment in the region has deteriorated; for example, serious soil erosion, sharp reductions in forest resources, and soil pollution caused by deposit mining occur frequently, and it is necessary to evaluate the ecological environmental risk posed by soil in the area.

### 2.2. Sample Collection

The study was completed on the basis of the national multipurpose soil survey. Surface soil samples were collected at a density of one sample/km^2^ from a sampling depth of 0–20 cm, and a total of 28,095 surface soil samples were collected. The collected soil samples were sun-dried, ground, and sieved through a +20 mesh sieve, and the sample combination was carried out using a 2 km × 2 km grid. A total of 7309 combined samples were obtained in this study. After the combined samples were mixed, approximately 30 g of sample was removed and used for pH analysis. Approximately 80 g of sample was taken and dried in an oven at 60 °C. Then, a pollution-free planetary ball mill was used to crush the sample to −200 mesh particle size to create an analytical sample that was sent to the laboratory for analysis. This evaluation work was carried out based on the analysis data of the 7309 surface soil samples that were obtained.

### 2.3. Analytical Methods and Quality Control

Soil samples were decomposed with hydrofluoric acid, nitric acid, and perchloric acid. After decomposition with aqua regia, the samples were moved to a plastic test tube, and then the volume was set and shaken. The clear solution was separated, and 3% nitric acid solution was used to dilute the solution 1000 times. Then, inductively coupled plasma-mass spectrometry (ICP-MS) was used to test the Cd, Cu, Ni, Pb, and Zn, and full-spectrum direct reading inductively coupled plasma-emission spectroscopy (ICP-OES) was used to complete the Cr test. Samples were decomposed with aqua regia, potassium permanganate solution was added for oxidation treatment, and the solution was diluted with oxalic acid solution. Then, potassium borohydride was used as a reducing agent to be pre-reduced by thiourea-ascorbic acid. Atomic fluorescence spectrometry (AFS) was used to measure the As with HG-AFS. Finally, the remaining solution was directly diluted to measure the Hg with CV-AFS using SnCl_2_ as a reducing agent.

Quality assurance and control measures were used throughout the testing process, and the national first-level standard substances (GBW07401–GBW07408, GBW07425–GBW07428) were used as the standard reference materials to monitor the testing process. During the analysis, four pieces of password samples were inserted for each batch (approximately 50 samples) for external and internal quality controls. Two blank tests were carried out in an analysis batch to control the blank changes. The results showed that the overall pass rate of the first-level standard substance samples for monitoring accuracy was 100%, the overall pass rate for the precision monitoring samples was 100%, the total element reporting rate was 99.97%, and the overall pass rate of randomly selected 5% repeatability test samples was 99.5%. The analysis results met the quality requirements of the technical specification for multipurpose regional geochemical survey DZ/T0258-2014 [31].

### 2.4. Assessment Methods of Heavy Metal Pollution in Soils

#### 2.4.1. Ecological Risk Index

In this study, the potential ecological risk index (*PERI*) was used to evaluate the potential ecological risk of heavy metals in soil. The *PERI* of a single heavy metal ($E_{r}^{i}$) and compound with eight heavy metals (*RI*) were calculated by Equations (1)–(3) [32]:

(1) $$C_{f}^{i}=\frac{C_{s}^{i}}{C_{n}^{i}}$$

(2) $$E_{r}^{i}=T_{r}^{i}\times C_{f}^{i}$$

(3) $$RI=\sum_{1}^{m}E_{r}^{i}$$

where $C_{f}^{i}$ is the pollution index of heavy metal *i*; $C_{s}^{i}$ is the test value of heavy metal *i* in the soil sample; $C_{n}^{i}$ is the background value of heavy metal *i*; $T_{r}^{i}$ is the biological toxicity response factor of each heavy metal; and the values of $T_{r}^{i}$ are listed in Table 1.

The *PERI*s of eight heavy metals, including As, Cd, Cr, Cu, Hg, Ni, Pb, and Zn, in soil were calculated. Hakanson [32] divided the ecological risk compound index (*RI*) into four risk levels: extremely strong risk (600 ≤ *RI*), strong ecological risk (300 ≤ *RI* < 600), moderate ecological risk (150 ≤ *RI* < 300), and low ecological risk (*RI* < 150), which helps researchers analyze the risk more intuitively.

#### 2.4.2. Noncarcinogenic Risk Assessment

The health risk assessment models provided by the USEPA were used in this study to evaluate noncarcinogenic risk [34,35,36]. This model considers different exposure pathways and the effects of different exposure frequencies based on different land uses. Adults in the living environment are mainly affected by the following three soil exposure pathways: (1) human beings ingest heavy metals through their daily diet; (2) inadvertent inhalation of heavy metals from surface dust and atmospheric dust; and (3) dermal absorption caused by contact with soil through production activities. The chronic daily intake of heavy metals under the three exposure pathways can be calculated as Equations (4)–(6) [7,36,37,38,39,40]:

(4) $$CDI_{ingest}=\frac{C_{soil}\times IR_{ing}\times ED\times EF}{BW\times AT}\times CF$$

(5) $$CDI_{inhale}=\frac{C_{soil}\times IR_{inh}\times ED\times EF}{PEF\times BW\times AT}$$

(6) $$CDI_{dermal}=\frac{C_{soil}\times SA\times SL\times ABS\times ED\times EF}{BW\times AT}\times CF$$

where $CDI_{ingest}$, $CDI_{inhale}$, and $CDI_{dermal}$ represent the chronic intake of heavy metals under three exposure pathways of ingestion, inhalation, and dermal contact, respectively, mg·(kg·d)^−1^; $C_{soil}$ is the concentration of heavy metals in the single pollution of surface soil, mg·kg^−1^; $IR_{ing}$ and $IR_{inh}$ are the weights of ingestion and inhalation soil intake, respectively, mg; all the parameters used in this study are shown in Table 2. The exposure frequency (*EF*) selects different *EF*s according to different land-use types in the study area: the farmland and residential land *EF* is 350 day·a^−1^; the industrial and mining land *EF* is 250 day·a^−1^; the forestland, grassland and other land-use type *EF* values are equal to 40 day·a^−1^ [36,38]. Exposure duration (*ED*) is divided into noncarcinogenic chronic risk and carcinogenic risk. The exposure period of noncarcinogenic chronic risk is 24 years [36], and the exposure period of carcinogenic risk is a lifetime. This study uses the average life expectancy of Yunnan Province in China calculated by the National Bureau of Statistics of China in 2019 of 69.5 years [41].

Heavy metals in the soil can be ingested by the human body through multiple exposure pathways, causing the risk of chronic diseases. It is defined as the hazard quotient (*HQ*) and characterized by the ratio of the amount of heavy metals ingested by the human body to the specified reference dose (*RfD*) of the pollutant under different exposure pathways. The hazard index (*HI*) is the sum of chronic risks. In this study, the *HI* represents the health risks of chronic diseases in humans caused by the three exposure pathways of the eight heavy metals, including As, Cd, Cr, Cu, Hg, Ni, Pb, and Zn. Chronic diseases may occur when the *HI* is greater than 1, and the risk of diseases increases with an increasing *HI* [36]. The *HQ* and *HI* of heavy metals can be expressed with Equations (7) and (8):

(7) $$HQ_{ij}=\frac{CDI_{ij}}{RfD_{ij}}$$

(8) $$HI=\sum_{j=1}^{n}\sum_{i=1}^{n}HQ_{ij}$$

where $CDI_{ij}$ represents the chronic daily intake of *i*th heavy metal through the *j*th exposure pathway, mg·(kg·d)^−1^; $RfD_{ij}$ represents the reference dose of *i*th heavy metal under the *j*th exposure pathway, mg·(kg·d)^−1^; and the reference dose corresponding to a single heavy metal used in this study is listed in Table 3.

#### 2.4.3. Carcinogenic Risk Assessment 

Soil carcinogenic risk is characterized by the probability that an individual will experience cancer through lifelong exposure to carcinogens in the environment, and this value is obtained by establishing a cancer slope factor (*CSF*) through the individual exposure to soil carcinogens by various exposure pathways [35], and the carcinogenic risk is calculated by Equation (9):

(9) $$Risk_{car}^{j}=CDI_{j}\times CSF_{j}$$

where $Risk_{car}^{j}$ is the carcinogenic risk from the *j*th exposure pathway, $CDI_{j}$ is the chronic daily intake of a carcinogen under the *j*th exposure pathway, mg·(kg·day)^−1^; and $CSF_{j}$ is the carcinogenic slope factor of the carcinogen under the *j*th exposure pathway. The carcinogenic slope factors used in this study are shown in Table 3, in which the skin absorption carcinogenic slope factors of Cd and Pb are calculated by dietary intake carcinogenic slope factors [36,50,51]. The calculation is shown in Equation (10). Finally, the comprehensive carcinogenic risk of a carcinogen was obtained by calculating the carcinogenic risk coefficient under different exposure pathways (Equation (11)) [40,52].

(10) $$CSF_{dermal}=\frac{CSF_{ingest}}{ABS_{GI}}$$

(11) $$Risk_{car}=Risk_{ingest}+Risk_{inhale}+Risk_{dermal}$$

where $ABS_{GI}$ is the gastrointestinal absorption factor, and the gastrointestinal absorption coefficients of Cd and Pb used in this study are 0.025 and 1.0, respectively [44]. Commonly, when the cancer risk coefficient ($Risk_{car}$) is lower than 1.0 × 10^−6^, which is equivalent to the probability of cancer occurring in one out of 1,000,000 people, the risk can be considered to be negligible, and when it is higher than 1.0 × 10^−4^, it is recognized that the carcinogenic risk is unacceptable [35,53]. Therefore, 1.0 × 10^−6^ has been recognized as the threshold for determining carcinogenic risk [54,55].

### 2.5. Positive Matrix Factorization Model (PMF)

The PMF is a reliable and effective pollution source identification method proposed by the USEPA [29]. It is used to calculate the source profile and source contributions of acceptor chemical components, and it can be expressed as follows:

(12) $$X_{ij}=\sum_{k=1}^{P}\left(g_{ik}f_{kj}+e_{ij}\right)$$

where $X_{ij}$ represents the concentration of *j*th element in the *i*th sample, mg·kg^−1^; $g_{ik}$ is the contribution of the *i*th sample from the *k*th source; $f_{kj}$ is the component of element *j* in source *k*; $e_{ij}$ is the residual matrix; and *P* is the number of factors. In addition, the objective function *Q* by the PMF model can be expressed as follows:

(13) $$Q_{i}=\sum_{j=1}^{n}\sum_{i=1}^{m}{\left(\frac{e_{ij}}{u_{ij}}\right)}^{2}$$

where $u_{ij}$ is the uncertainty of the *j*th element in the *i*th sample, which is calculated by the concentration and method detection limit (*MDL*). The concentration of each heavy metal in this study is greater than the *MDL*; therefore, the uncertainty can be expressed as follows:

(14) $$u_{ij}=\sqrt{{\left(\delta \times C\right)}^{2}+{\left(0.5\times MDL\right)}^{2}}$$

where δ is the relative deviation, generally 5% [8,29,56,57]; *C* is the concentration of element *j*, mg·kg^−1^; and *MDL* is the method detection limit.

### 2.6. Statistical Analysis

Statistical analysis of characteristic parameters was performed by SPSS (version 19.0) (IBM, Almonk, NY, USA) software. The spatial distributions of heavy metal concentrations adopted the power exponential weighting method using the software GeoIPAS (version 3.2) (Jinwei Graphic Information Technology Co., Ltd., Urumqi, China), and the ecological health risks were determined using the software ArcGIS (version 10.2) (Esri, Redlands, CA, USA). The identification of heavy metal sources was performed using PMF (version 5.0) (USEPA, Washington, DC, USA).

## 3. Results and Discussion

### 3.1. Soil Properties and Metal Accumulation in Soils

**Figure 2:**
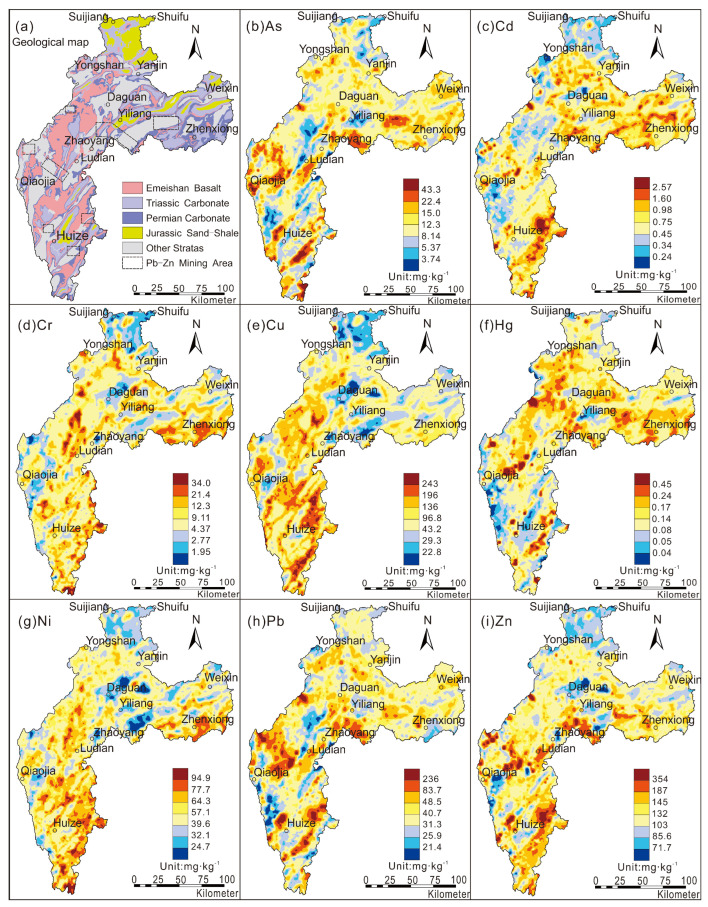
Spatial distribution of heavy metal concentrations in the study area. (**a**) geological map, (**b**) As, (**c**) Cd, (**d**) Cr, (**e**) Cu, (**f**) Hg, (**g**) Ni, (**h**) Pb, (**i**) Zn.

According to the statistical results of soil heavy metals in the study area (Table 4), these eight heavy metals showed enrichment, except for As, relative to the background value of soil (layer A) in China [14,15]. The background values of heavy metals Cu and Cd are 73.6 mg·kg^−1^ and 0.56 mg·kg^−1^, respectively, and their enrichment coefficients are 3.2 and 5.8, respectively, which are values indicative to the typical soil heavy metal enrichment area in southwest China. The coefficients of variation of Cu, Hg, Cd, and As in the area are 72%, 53%, 50%, and 48%, respectively, with a wide variation range, which shows that the sources of these heavy metals are complex. Based on the ratio of geochemical background value to reference value (*K_background_/_reference_*) in the study area, it is clear that As, Cd, Hg, and Pb are strongly enriched in the surface soil relative to the deep soil, and more than 80% of the surface soil in the study area displays enriched Cd; compared to the deep soil, the surface soil enrichment degree is 3.5 times greater. These characteristics indicate that most of the heavy metals in the surface soil are affected by natural or human activities during the process of soil formation. As a result, the distribution pattern of heavy metals in the surface soil changes, especially the biophilic elements and environmental elements, which are the most significantly affected. These elements show the characteristics of regional enrichment, while others show local enrichment or depletion affected by fractional factors (Figure 2). The heavy metal characteristics in the soil of areas with high geochemical background levels are related to the inheritance of the parent material and driven by the element geochemical driving mechanism and the distribution of typical parent material of carbonate rock [16,19,58,59]. However, the widely distributed lead–zinc deposits (spots) in the area have become another main source of heavy metals in the soil, and the spatial distribution of As, Pb, and Cd shows enrichment characteristics in the mining area (Figure 2). Therefore, it is believed that the heavy metals in the surface soil of the study area have multiple sources, and mining is one of them.

**Table 4:** Descriptive statistics for heavy metal concentrations in surface soil.

Items	As	Cd	Cr	Cu	Hg	Ni	Pb	Zn
	*wt*/mg·kg^−1^							
Arithmetic Mean	11	0.64	122	95.4	0.13	53	37	122
Std.deviation	5.26	0.32	47.0	68.9	0.07	19.1	10.8	32.2
Coefficient of variation (%)	48	50	39	72	53	36	29	26
Maximum	26.7	1.60	262	302	0.32	110	69.2	218
Minimum	1.44	0.04	15.4	3.16	0.02	4.10	8.89	27.3
Geometric Mean	10.3	0.64	119	72.3	0.11	50.2	37.4	120
Background value	10.1	0.56	109	73.6	0.11	52.5	35.1	122
Background value of Chinese soil [14,15]	11.2	0.10	61.0	23.0	0.07	27.0	26.0	74.0
Enrichment coefficient (Dimensionless)	0.90	5.77	1.79	3.20	1.72	1.94	1.35	1.65

### 3.2. Heavy Metal Pollution Assessment in Soils

#### 3.2.1. Potential Ecological Risk of Heavy Metals in Soil

**Figure 3:**
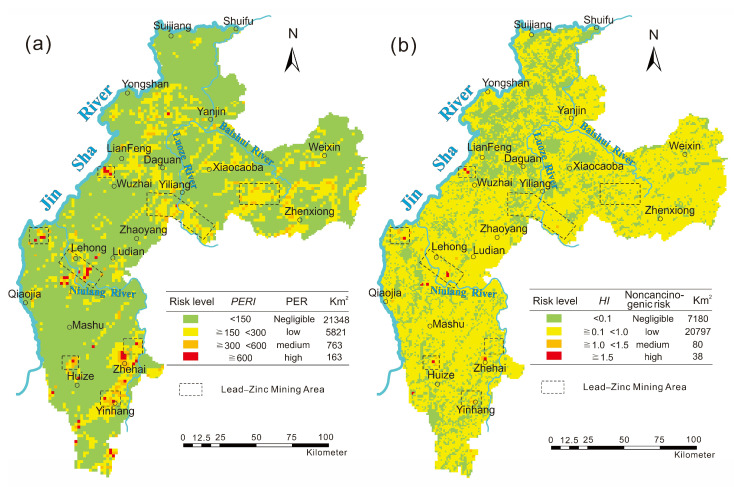
Spatial distribution of the compound ecological risk index (*RI*) (**a**) and hazard index (*HI*) (**b**) of heavy metals in surface soil.

The *PERI*s of heavy metals in 28,095 sampling sites of surface soil were calculated and are shown in Figure 3a, which shows that 76% of the study area has a low ecological risk (*RI* < 150); moderate ecological risk areas (150 ≤ *RI* < 300) account for 20.7% of the study area and are mainly distributed in Huize, Ludian, Yongshan, Yiliang, and Zhenxiong; high ecological risk areas (300 ≤ *RI* < 600) account for 2.72% of the study area and are interspersed with moderate-risk areas; extremely high ecological risk areas (600 ≤ *RI*) account for less than 1% of the study area and can be found in Huize, Ludian, and Yongshan. It is worth noting that the distribution of extremely high ecological risk areas is consistent with lead–zinc mining in the study area (Figure 3a). It is believed that the extremely high potential ecological risks of heavy metals in the soil are mainly caused by mining activities.

**Figure 4:**
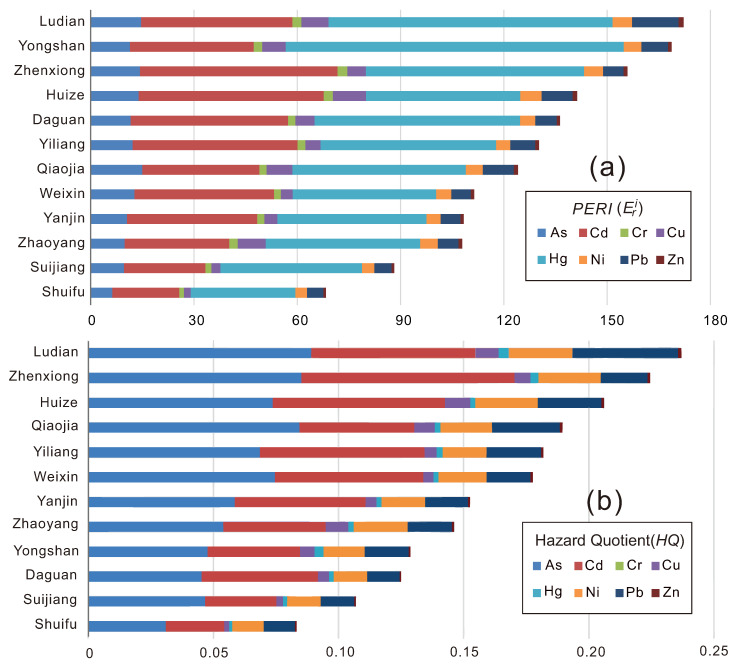
Average values of the potential ecological risk index ($E_{r}^{i}$) (**a**) and hazard quotient (*HQ*) (**b**) of heavy metals in the study area.

The distribution of the single indicator *PERI* ($E_{r}^{i}$) in each county shows that the largest contribution to the compound ecological risk (*RI*) is Hg ($E_{r}^{Hg}$), followed by Cd ($E_{r}^{Cd}$) and As ($E_{r}^{As}$) (Figure 4a). Hg, Cd, and As are the main elements that cause potential ecological risks in soil. The contribution rates of the eight heavy metals to ecological risks followed a descending order: Hg > Cd > As > Pb > Cu > Ni > Cr > Zn (Figure 4a). Ludian and Yongshan have the highest risk of Hg pollution, while the risk of Cd mainly affects Zhenxiong and Huize County, and the risk of As is concentrated in Qiaojia, Ludian, and Zhenxiong. The average value of the compound ecological risk indexes (*RI*) in Ludian, Yongshan, and Zhenxiong is higher than 150, which shows a high potential ecological risk of heavy metals in these three counties. The *RI* values of the other counties are all greater than 100 except for that in Suijiang and Shuifu (Figure 4a), indicating that the total amount of heavy metals in the soil is relatively high and that there is serious potential ecological risk in the study area.

**Figure 5:**
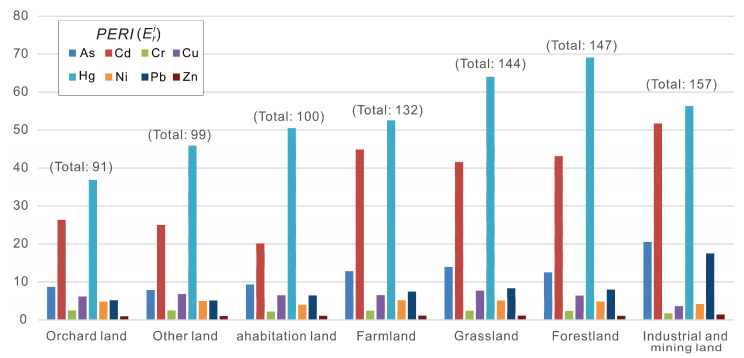
Average ecological risk index ($E_{r}^{i}$) of heavy metals in different land uses.

The potential ecological risk of heavy metals in the soil of the study area has obvious differences between various land-use types (Figure 5). Figure 5 shows that the soil of industrial and mining land has the highest ecological risk, and the total ecological risk indexes of the eight heavy metals exceed 150, followed by that of forestland and grassland. Hg, Cd, and As are still the major elements causing risk. Among these heavy metals, Hg shows a high risk in all land-use types, especially forestland and grassland ($E_{r}^{Hg}\;$ > 60); the risk of Cd is relatively high in the soil of industrial land, mining land, and farmland, followed by forestland and grassland. The soil in these three land-use types has frequent human activities, and soil from forestland and grassland is generally characterized by the accumulation of organic matter. Both of these characteristics explain the accumulation of heavy metals in soil [19], and it has been shown that the Cd ecological risk is affected not only by human activities but also by the adsorption of organic matter in the surface soil. The ecological risk of heavy metal As is more significant in the soil of industrial and mining land, indicating that its source is mainly related to the lead–zinc mine in the area. Heavy metals show different levels of potential ecological risks in different land-use types, and the source of heavy metals in the area is complex and more or less restricted by types of land use. More importantly, the impact of land-use types on ecological risk assessment in the area cannot be ignored.

#### 3.2.2. Noncarcinogenic Risk Assessment

The exposure of heavy metals in soil can be characterized by the chronic daily intake (*CDI*), mg·(kg·day)^−1^. The results of several studies on the accumulation of heavy metals in soils under different land uses show that the diversity of land-use patterns directly affects the exposure frequency and *CDI* of adults exposed to the soil environment, which leads to unreasonable evaluation results of human health risks under different exposure methods [25,26]. The land-use types in the study area are mainly forestland, farmland, and grassland, and there are a small number of paddy fields, orchard land, urban land, and industrial and mining land scattered throughout the area. This study selected different exposure pathways according to the production and living conditions of adults under different land-use types. Agricultural land and residential land *EF* is 350 day·a^−1^; industrial and mining land *EF* is 250 day·a^−1^; and forestland, grassland, and other land-use type *EF* values are equal to 40 day·a^−1^ [36,38]. The *CDI*s of eight heavy metals were calculated through dietary intake, inadvertent inhalation, and dermal contact, and the calculation results of the hazard quotient (*HQ_ij_*) and the hazard index (*HI*) of 28,095 samples were based on the known reference dose (*RfD*), mg·(kg·day)^−1^ [44,46,47].

As seen from Figure 3b, the noncarcinogenic chronic risk of soil in the study area is generally shown as low risk (0.1 ≤ *HI* < 1.0), accounting for 74% of the study area; moderate-risk areas (1.0 ≤ *HI* < 1.5) are distributed in Huize, Ludian, and Yiliang, accounting for 0.28% of the study area. Huize and Ludian are the main areas with moderate risk, which may be related to the widespread existence of Cd-rich strata causing the accumulation of the heavy metal Cd in the soil. High-risk areas (1.5 ≤ *HI*) are scattered in Huize, Ludian, and Daguan counties, of which the Ludian–Lehong and Zhehai areas are more concentrated (Figure 3b); these counties are areas with concentrated lead–zinc mines, indicating that the mining process is one of the important causes of health risks posed by the soil.

As has the largest contribution to human noncarcinogenic chronic risk, with an average contribution of more than 35%, followed by that of Cd and Pb. The contribution rates of the eight heavy metals to the *HQ* followed a descending order: *HQ_As_* > *HQ_Cd_* > *HQ_Pb_* > *HQ_Ni_* > *HQ_Cu_* > *HQ_Hg_* > *HQ_Zn_* > *HQ*_Cr_ (Figure 4b). The contribution order of heavy metals to the *HI* under the three exposure pathways was *HQ_dermal_* > *HQ_ingest_* > *HQ_inhale_*, where the average value of *HQ_dermal_* exceeded 55%, which was the main exposure pathway in which adults were exposed to heavy metals in the study area. Among the 12 counties in the study area, the average human health risk index values in Ludian, Zhengxiong, and Huize were all greater than 0.2, indicating a higher risk; the values in Shuifu were relatively low (*HI* < 0.1), and the remaining counties had values between 0.1 and 0.2 (Figure 4b).

#### 3.2.3. Carcinogenic Risk Assessment

**Figure 6:**
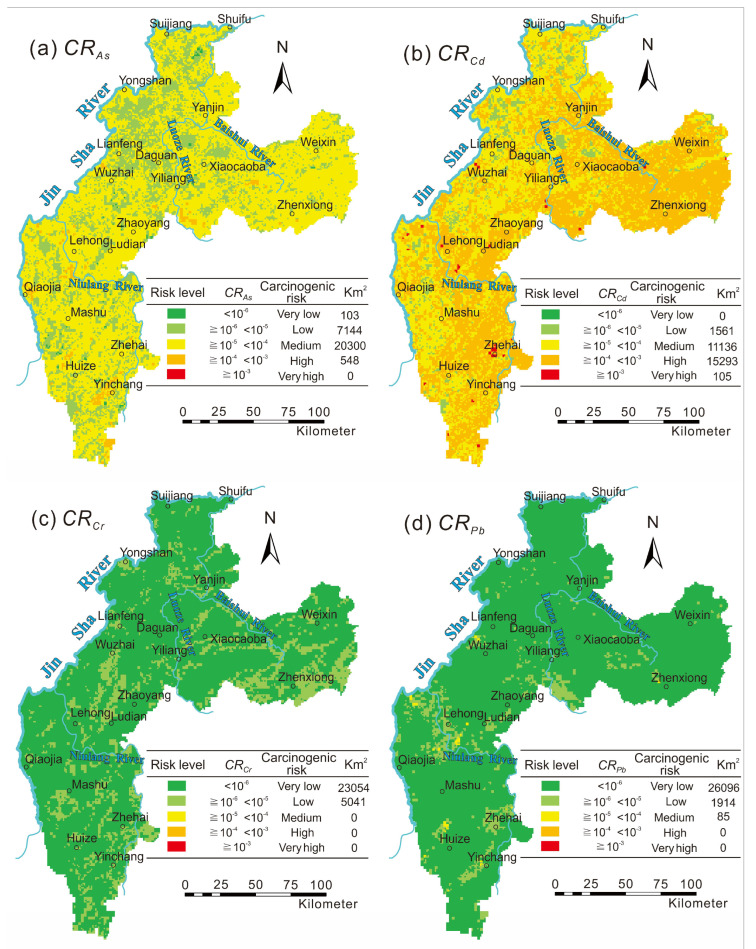
Spatial distribution of carcinogenic risk (*CR*) upon exposure to residents in the study area. (**a**) *CR_As_*_,_ (**b**) *CR_Cd_*, (**c**) *CR_Cr_*_,_ (**d**) *CR_Pb_*.

This study conducted a carcinogenic risk assessment of the heavy metals As, Cd, Cr, and Pb in the surface soil. The remaining heavy metals were not evaluated in this study since there is no cancer risk factor reported. The carcinogenic risk of As was moderate, accounting for 72.3% of the research area. The high-risk areas were mainly concentrated in Huize, Yinchang, Wuxing, Lehong, Yiliang, Maoping, and Wanchang, accounting for 2% of the research area (Figure 6a). The high carcinogenic risk of As was consistent with the distribution of lead–zinc mines in the study area, indicating the risk was mainly caused by lead–zinc mining activities. The carcinogenic risk of Cd was mainly high risk, accounting for 54.4% of the research area. The high-risk areas were mainly distributed in Zhenxiong, Weixin, Huize, and Ludian, and the extremely high-risk areas were distributed in Yinchang, Lehong, and Yi Liang (Figure 6b), which was the result of the simultaneous actions of Permian carbonate and lead–zinc mining activities [16]. The carcinogenic risk index of Cr and Pb was less than 1.0 × 10^−4^, and the whole area showed negligible risk (Figure 6c,d). Cr and Pb was enriched in the soil of the study area, but duo to the lack of a reported *CSF* of Cr and the *CSF* of Pb was 1–2 orders of magnitude lower than the other elements [44], it showed a low biological carcinogenic risk, which resulted in their negligible carcinogenic risk.

The carcinogenic risk of four heavy metals in the study area was Cd > As > Pb > Cr in the order of risk degree, and the carcinogenic risk of different counties differed significantly in spatial distribution. The carcinogenic risk of Cd mainly affected Zhenxiong, Ludian, Huize, and Weixin, and more than 60% of the soil in the four counties had a high carcinogenic risk, especially in Zhenxiong, which was as high as 84.7%. It is worth noting that there were few areas with a very high risk of Cd in this region, and all counties had areas less than 1% except Ludian, which reached 1.1%. The carcinogenic risk of the heavy metal As in the soil mainly affected Zhenxiong, Weixin, and Ludian counties; more than 80% of the soil in the three counties showed a medium carcinogenic risk, of which the high risk areas of Qiaojia, Huize, and Yiliang exceeded 2%. In summary, the soil of some counties in the study area had a higher risk of carcinogenesis by heavy metals; however, the heavy metals in the soil that cause these risks have multisource characteristics.

### 3.3. Source Identification of Metals by PMF

**Figure 7:**
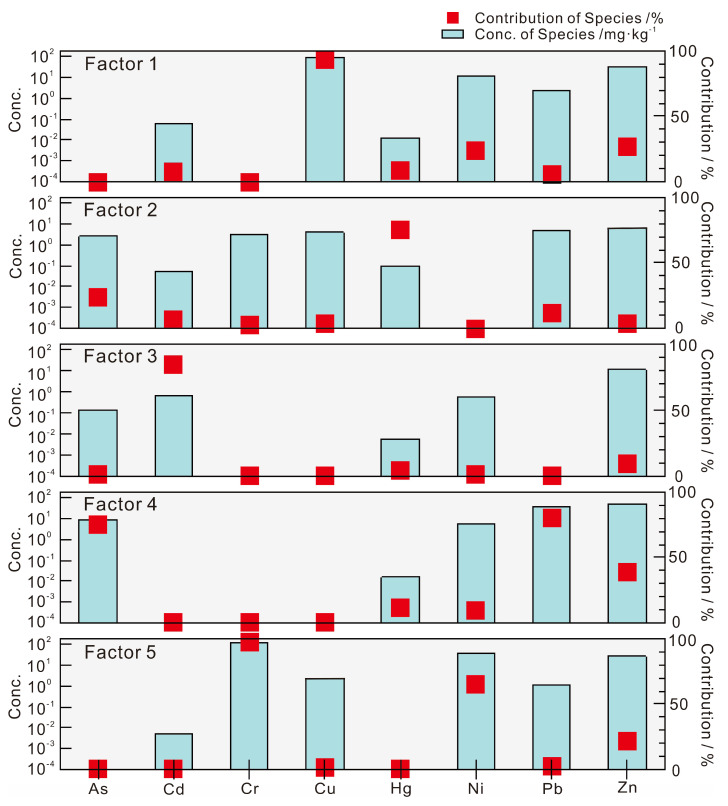
Factor profiles and source contributions of heavy metals from the PMF model.

The distribution of potential ecological risks and human health risks shows that the soil heavy metals have multiple sources. To further determine the source of heavy metals and the contribution of each heavy metal to the pollution source in the surface soil of the study area, this research used the PMF [29] to identify the source of the eight heavy metals. All heavy metals were set to be strong according to the signal-to-noise ratio (S/N) to ensure the rationality of the model during the identification process; additionally, the operating factors were selected as 3, 4, and 5, and the running number was set to 20. The goodness-of-fit *Q* (robust) and *Q* (true) values obtained from the PMF model were the smallest and most stable when the number of running factors was 5, and the correlation coefficient between the observed and predicted concentration value (*r*^2^) of each heavy metal was the largest, which indicated that these five factors could comprehensive and reasonably reflect the information in the original data [8]. Figure 7 shows the analysis results for the eight heavy metals in the study area, and it is clear there are five main sources of heavy metals in the study area. These sources have different degrees of impact on the heavy metals in the surface soil.

Factor 1 has higher factor contributions of Cu, Zn, and Ni (contribution rates are 93.6%, 26.5%, and 23.8%, respectively). Over 20% of the study area has exposed basalt, and the distribution range of Cu and Ni anomalies in the soil is consistent with the widely exposed basalt in the area (Figure 2). Existing studies show that the spatial distribution pattern of Cu, Ni, and Zn in soils of southwestern China is mainly controlled by the distribution of Emeishan basalt [16,20], and the main morphological components of Cu and Ni in surface soil are in the residual fractions [19,60], which indicates that Cu, Ni, and Zn in the surface soil are inherited from the basalt parent rock. Therefore, Factor 1 is interpreted as a natural source caused by the geological background of Emeishan basalt in the study area.

Factor 2 is mainly related to Hg, As, and Pb (contribution rates are 75.6%, 23.9%, and 11.7%, respectively). Studies have shown that over 40% of Hg emissions in China are from anthropogenic sources caused by coal combustion [61], and atmospheric deposits produced by coal combustion are the main source of heavy metal As [62]. The research area of Zhaotong city is located at the border of Yunnan and Guizhou Provinces, which represent the main areas of coal production in southwest China. There are a large number of brown coal mines in Zhenxiong and Weixin in the district [63,64]. Slime, which is a mixture of coal, clay, and water, is the main domestic fuel in these rural areas, and a large amount of smoke dust is generated by coal combustion and residual coal ash and poured into the soil, causing the accumulation of heavy metals in soil. It is generally believed that the source of Pb is the emissions of automobile exhaust that enters the soil through atmospheric deposition; this Pb can remain in the soil environment for a long time [61,65]. It could be concluded that Factor 2 indicates a comprehensive source of pollution caused by atmospheric subsidence of coal burning and traffic emissions.

Factor 3 has the strongest correlation with Cd (the contribution rate is 85%), and the remaining heavy metals contribute less than 10% to this factor. Compared with the background value of soil (layer A) in China [14,15], Cd is the most enriched heavy metal in the study area, with an enrichment coefficient as high as 5.77. Ma et al. [59] believe that the accumulation of Cd in the soil of southwest China is mainly related to the exposed carbonate rocks in the area, and the spatial distribution of Cd in the study area suggests the same conclusion (Figure 2). Many research results have shown that the source of soil Cd is mainly related to agricultural production, such as pesticides and chemical fertilizers [66,67,68]; however, this study was not conducted on agricultural land, and 17.1% of all 28,095 soil samples that were collected were from forestland and grassland, which had no agricultural activity. A total of 40.2% of the samples came from the area where carbonate is exposed, in which the heavy metal Cd is highly enriched. In addition to Cd, there is no other heavy metal contribution in Factor 3; therefore, Factor 3 is considered to be the source of Permian carbonate rock.

Factor 4 is dominated by Pb, As, Zn, and Hg (contribution rates are 79.7%, 74.9%, 38.5%, and 11%, respectively). Commonly, soil from lead-zinc mining areas is enriched with Pb, Zn, and As from the mining of deposits [69,70,71,72,73]. In addition to the influence of parent materials such as basalt and carbonate rock, the medium–large lead–zinc mines distributed in the area have become an important main source of heavy metals in the soil. According to incomplete statistics, 86 lead–zinc mines have been identified in the study area, including 9 large mines. The proven reserves of lead–zinc mines in Zhaotong city of Yunnan Province exceed 700 × 10^4^ tons, and Zhaotong and Qujing are the third and fourth largest cities, respectively, of lead and zinc resource reserves in Yunnan Province [74]. The spatial distribution of Pb, Zn, and As in the soil in the study area is consistent with the distribution of these known lead–zinc mining areas (Figure 2), which are mainly distributed in Yiliang, Zhenxiong, Qiaojia, and Huize, and these areas are also high-risk areas with soil that can potentially harm ecological and human health (Figure 3). Therefore, it is believed that Factor 4 mainly reflects the anthropogenic influence source of heavy metal accumulation caused by lead–zinc deposit mining activities.

Cr, Ni, and Zn are associated with Factor 5 (contribution rates are 97.4%, 65.5%, and 21.3%, respectively). Some researchers believe that the amounts of Cr, Ni, and Zn in the soil are controlled by the diagenetic parent rock and are related to the diagenetic composition [11,68]. The soil formed by weathered sediments of Triassic sedimentary rock in the study area showed more Cr concentrations than did the other sediments, even more than that in areas with basalt weathering, which accounted for 19.3% of the total number of samples. The inconsistent melting of orthopyroxene during the formation of the Emeishan basalt in the upper mantle is accompanied by Triassic volcanic activity, which leads to the depletion of the strong compatible element Cr in the basalt and the enrichment in the Triassic sedimentary rocks [75]. Furthermore, the results of soil morphological analysis of Cr, Ni, and Zn show that more than 80% of its composition is in the residual fractions [19,59], indicating that the source of heavy metals is mainly related to the parent rock. Therefore, it could be inferred that Factor 5 reflects the information of Triassic carbonate rocks in the study area.

**Figure 8:**
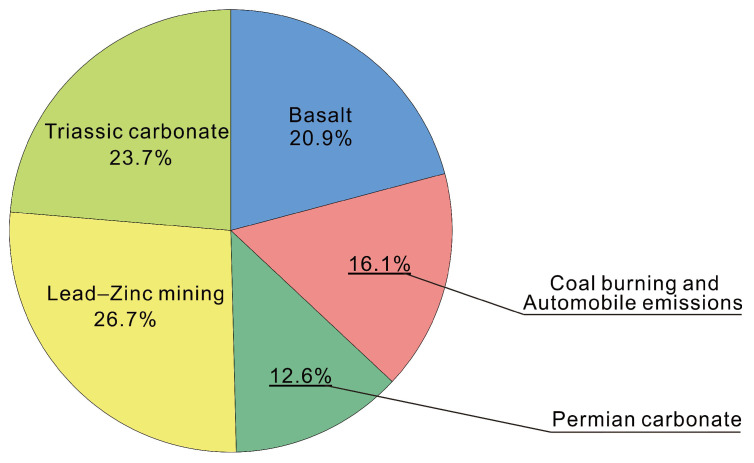
Contributions rates of different sources by PMF.

In summary, the sources of heavy metals in the study area are mainly reflected by the above five factors, of which Factor 1, Factor 3, and Factor 5 are all natural sources, indicating information on the basalt and carbonate rocks in the study area; in contrast, Factor 2 and Factor 4 reflect anthropogenic sources, which indicate information on mining activities and atmospheric precipitation caused by coal burning and traffic emissions. The total contribution of the five factors followed a descending order: Factor 4 > Factor 5 > Factor 1 > Factor 2 > Factor 3 (Figure 8). Lead–zinc deposit mining activities have become the largest source of heavy metals in the study area, the contributions of other factors of heavy metal sources are relatively balanced. Factor 1, Factor 3, and Factor 5 are all natural sources, mainly caused by the geological background, but the higher carcinogenic risk that was identified deserves more attention. It is necessary to select several typical areas to strengthen the detailed investigation and the safety evaluation of agricultural products. This will help to obtain and understand the source and evolution information of soil heavy metals in local area.

## 4. Conclusions

Heavy metals show different levels of potential ecological risks in different land-use types, and the source of heavy metals in the area is complex and more or less restricted by types of land use. More importantly, the impact of industrial and mining land and farmland on ecological risk assessment is significant and it cannot be ignored.

Mining activities were one of the important factors affecting health risks posed by the soil. The contribution of the eight heavy metals to the *HQ* followed a descending order: As > Cd > Pb > Ni > Cu > Hg > Zn > Cr, and the three exposure pathways showed the following: *HQ_dermal_* > *HQ_ingest_* > *HQ_inhale_*. Cd and As are the main contributors to chronic health and carcinogenic risk in the study area; the heavy metals in the soil that cause these risks have multisource characteristics.

The soil heavy metals in the study area mainly comes from five sources, of which parent material such as basalt, Permian, and Triassic carbonate rock constitutes the natural geological background source, the mining activities of lead–zinc mines and the emissions of coal burning and automobile exhaust from residents constitute an anthropogenic source. The accumulation of heavy metals by mining activities contributed the most to the soil. The total contribution of the five sources followed a descending order: lead–zinc mining (26.7%) > Triassic carbonate (23.7%) > basalt (20.9) > coal burning and automobile emissions (16.1%) > Permian carbonate (12.6%).

The ecological risk assessment of areas with high geochemical background should focus on the analysis and understanding of the geological background, combined with the comprehensive judgment of the results of pollutant source analysis. Based on the different exposure pathways, the impact of human activities on health risk assessment is crucial. These results provide basic information on the study of heavy metals and guidance on the selection of natural source treatment options in the high geochemical background area of southwest China.

## Figures and Tables

**Table 1:** Reference values of biological toxicity response factors of heavy metals.

Heavy Metals	As	Cr	Cd	Cu	Pb	Ni	Zn	Hg	References
Toxic response factors	10	2	30	5	5	5	1	40	[32,33]

**Table 2:** Exposure parameters used to characterize the chronic daily intake (*CDI*) of heavy metals.

Symbol	Parameter		Value	Unit	References
*IR_ing_*	Ingestion rate		100	mg·day^−1^	[36]
*IR_inh_*	Inhalation rate		12.8	m^3^·day^−1^	[42]
*EF*	Exposure frequency	Farmland and residential land	350	day·a^−1^	[36]
		Industrial and mining land	250		[38]
		Forestland and other types	40		
*ED*	Exposure duration	Non-carcinogenic risk	24	a	[36]
		Carcinogenic risk	69.5		[41]
*SA*	Exposed skin area		5700	cm^2^	[36]
*SL*	Skin adherence factor		0.07	mg·(cm^2^·day)^−1^	[36]
*ABS*	Dermal absorption factor		0.03 (As), 0.001 (Cd), 0.04 (Cr), 0.10 (Cu), 0.05 (Hg), 0.35 (Ni), 0.006 (Pb), 0.02 (Zn)	unitless	[43,44]
*PEF*	Particle emission factor		1.36 × 10^9^	m^3^·kg^−1^	[36]
*AT*	Average exposure time		*ED* × 365	days	[36]
*BW*	Average bodyweight		62	kg	[45]
*CF*	Conversion factor		1.00 × 10^−6^	unitless	[42]

**Table 3:** Reference dose (*RfD*) and carcinogenic slope factor (*CSF*) of heavy metals for three exposure pathways.

Heavy Metals	*RfD*/mg·(kg·d)^−1^			*CSF*/mg·(kg·d)^−1^		
	*RfD_ingest_*	*RfD_inhale_*	*RfD_dermal_*	*CSF_ingest_*	*CSF_inhale_*	*CSF_dermal_*
As	3.00 × 10^−4^ [44]	1.50 × 10^−5^ [44]	1.23 × 10^−4^ [46]	1.50 × 10^0^ [44]	1.51 × 10^1^ [46]	1.50 × 10^−0^ [47]
Cd	1.00 × 10^−3^ [44]	1.00 × 10^−5^ [44]	1. × 10^−5^ [46]	6.10 × 10^0^ [48]	1.47 × 10^1^ [47]	2.44 × 10^2^ *
Cr	1.50 × 10^0^ [42]	1.50 × 10^0^ **	1.50 × 10^0^ **		4.20 × 10^1^ [46]	
Cu	4.00 × 10^−2^ [44]	4.00 × 10^−2^ **	1.20 × 10^−2^ [46]			
Hg	3.00 × 10^−4^ [44]	3.00 × 10^−4^ [44]	2.10 × 10^−5^ [46]			
Ni	2.00 × 10^−2^ [44]	9.00 × 10^−5^ [44]	5.40 × 10^−3^ [46]			
Pb	3.50 × 10^−3^ [46]	3.50 × 10^−3^ **	5.25 × 10^−4^ [46]	8.50 × 10^−3^ [49]	4.20 × 10^−2^ [47]	8.50 × 10^−3^ *
Zn	3.00 × 10^−1^ [44]	3.00 × 10^−1^ **	6.00 × 10^−2^ [46]			

* Get by *CSF_dermal_* = *CSF_ingest_*/*ABS_GI_* [44]; ** Use the *RfD_ingest_* instead.

## Data Availability

The data presented in this study are available on request from the corresponding authors. The processed data are not publicly available as the data also form part of an ongoing study.
